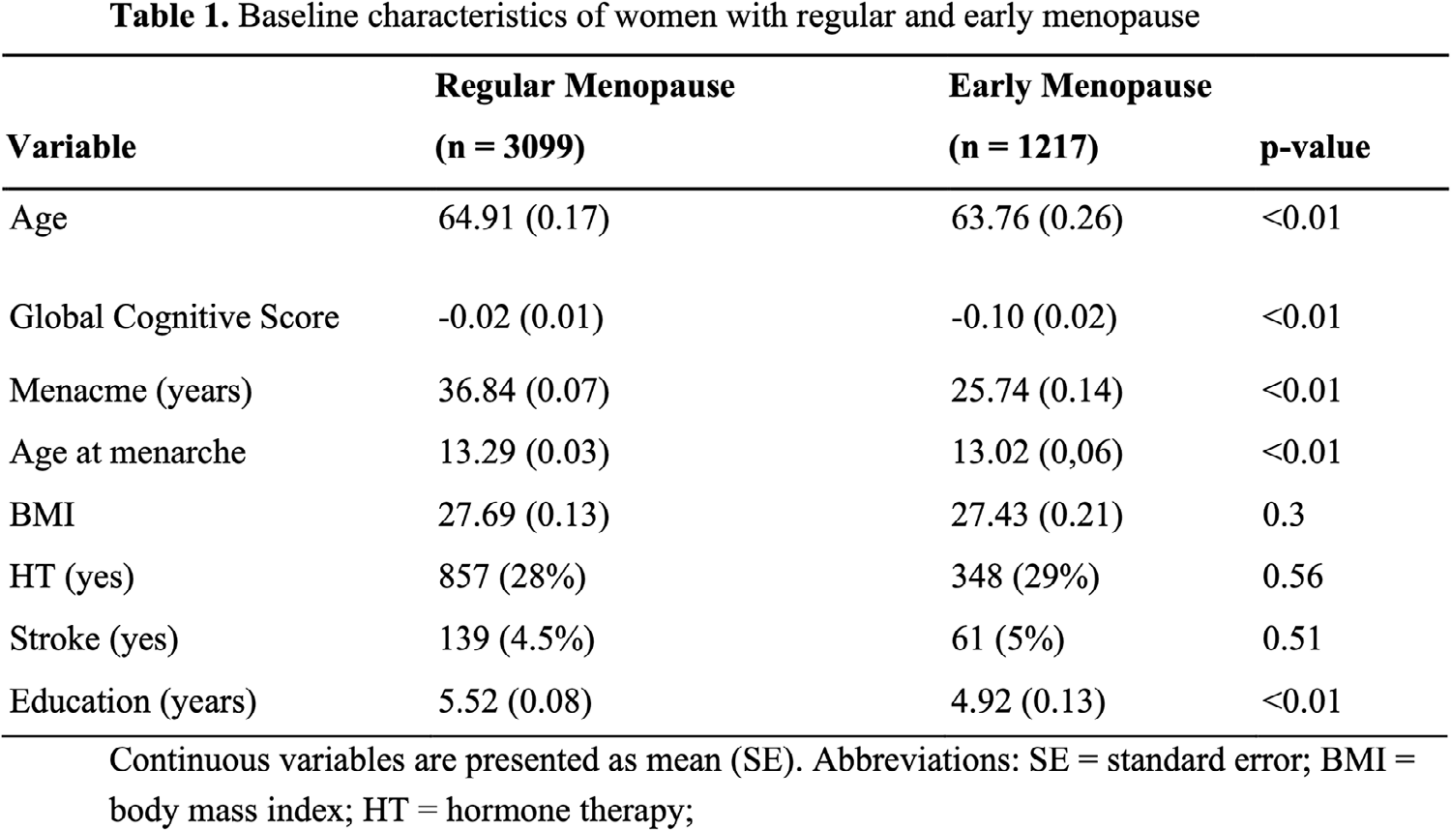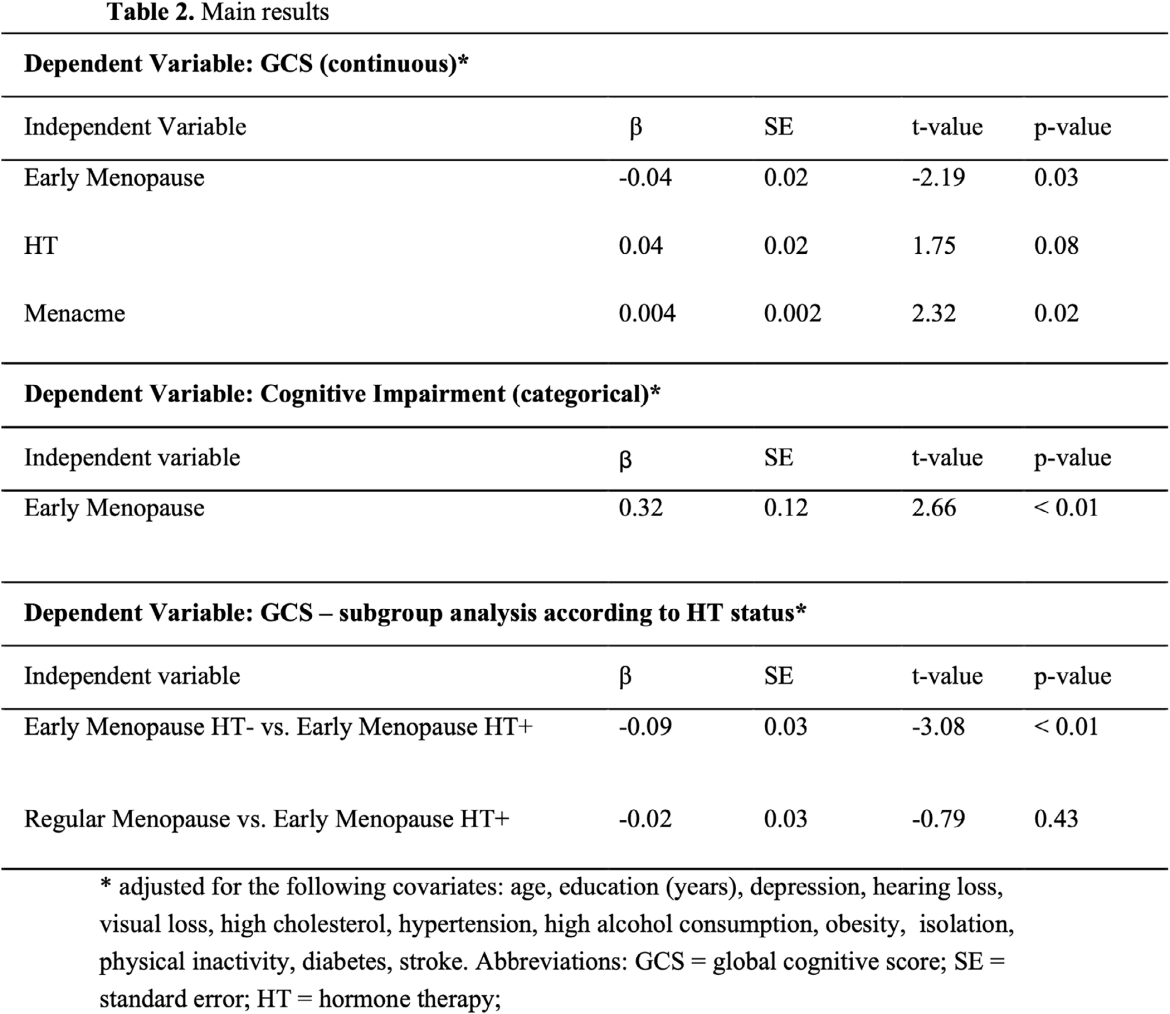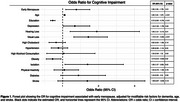# Early Menopause as a Modifiable Sex‐Specific Risk Factor for Cognitive Impairment: Findings from the ELSI‐Brazil Study

**DOI:** 10.1002/alz70860_106255

**Published:** 2025-12-23

**Authors:** Alvaro de Oliveira Franco, Eduardo R. Zimmer, Raphael Machado Castilhos

**Affiliations:** ^1^ Hospital de Clinicas de Porto Alegre, Porto Alegre, Rio Grande do Sul, Brazil; ^2^ Universidade Federal do Rio Grande do Sul, Porto Alegre, Rio Grande do Sul, Brazil

## Abstract

**Background:**

Early menopause (EM) is a potential risk factor for cognitive impairment, yet its impact remains underexplored in low‐ and middle‐income countries, where modifiable risk factors play a key role in dementia development. This study investigates how EM, hormone therapy (HT), and reproductive lifespan influence cognitive outcomes in Brazilian women.

**Methods:**

Data were analyzed from 4,316 postmenopausal women (≥50 years) in the Brazilian Longitudinal Study of Aging (ELSI‐Brazil). EM was defined as onset before 45 years. Cognitive performance was assessed via a global cognitive score (GCS), calculated from Z‐scores of five cognitive subdomains: orientation, fluency, episodic memory, semantic memory, and prospective memory. Covariates included age, previous stroke, and modifiable dementia risk factors: education, depression, sensory deficits, and metabolic factors. Linear regression modeled GCS, while logistic regression estimated the odds of cognitive impairment (GCS ≤ −1.5 SD). Subgroup analyses stratified women by EM and HT use. T‐tests and chi‐squared tests were used when appropriate. All statistical analyses were performed in R, with *p* < 0.05 considered statistically significant.

**Results:**

Baseline characteristics of EM (*n* = 1,217) and regular menopause (*n* = 3,099) groups are displayed in Table 1. EM was independently associated with lower GCS (β: −0.04, *p* = 0.03), while years of menacme (β: 0.004, *p* = 0.02) were protective. HT was not significantly associated with GCS (β: 0.04, *p* = 0.08) (Table 2). EM increased the odds of cognitive impairment (OR: 1.38, 95% CI: 1.09–1.76) (Figure 1). Subgroup analyses showed EM/HT− had significantly lower GCS than EM/HT+ (β: −0.09, *p* < 0.01), while EM/HT+ was comparable to regular menopause groups (*p* = 0.43) (Table 2). Moderation analysis confirmed a significant interaction between HT and EM (β: 0.11, *p* = 0.02), suggesting HT attenuates EM's negative impact.

**Conclusions:**

EM is an independent risk factor for cognitive impairment, while a longer reproductive lifespan appears to be associated with cognitive health. The interaction between EM and HT highlights HT's potential to mitigate cognitive decline, supporting EM as a modifiable sex‐specific risk factor for cognitive impairment.